# Supplementary material for: Protein Kinase A (PRKA) Activity Is Regulated by the Proteasome at the Onset of Human Sperm Capacitation
Source: Cells. 2021 Dec 11;10(12):3501. doi: 10.3390/cells10123501 (PMC8700002; doi:10.3390/cells10123501)
Supplement: Supplementary file 1 [file cells-10-03501-s001.zip › cells-1467913-supplementary.pdf]

Table.S1 Semen quality parameters of ejaculates used in the study

Parameters	*Reference limit (WHO)	Semen sample **(n=27)
Sperm concentration	≥15 x 10^6/mL	81 ± 29
Total sperm count in ejaculate	≥39 x 10^6	165 ± 21
Semen volume	≥1.5 mL	2.6 ± 1.3
Sperm motility (PR + NP, %)	≥ 40%	87 ± 7.8
Sperm progressive motility	≥ 32%	78 ± 10
Viability (live spermatozoa, %)	≥ 58%	91 ± 4.8
Semen pH	≥ 7.2	8.0 ± 1.5

PR: progressive motility; **NP:** non-progressive motility.
Data are presented as the mean + SEM.
*Lower reference limits of semen parameters were defined by the World Health Organization (2010) standards.
** Obtained from 20 healthy donors.
